# Ensemble deep learning for the prediction of proficiency at a virtual simulator for robot-assisted surgery

**DOI:** 10.1007/s00464-021-08999-6

**Published:** 2022-01-12

**Authors:** Andrea Moglia, Luca Morelli, Roberto D’Ischia, Lorenzo Maria Fatucchi, Valentina Pucci, Raffaella Berchiolli, Mauro Ferrari, Alfred Cuschieri

**Affiliations:** 1 EndoCAS, Center for Computer Assisted Surgery, University of Pisa, Edificio 102, via Paradisa 2, 56124 Pisa, Italy; 2 General Surgery Unit, Cisanello Teaching Hospital of Pisa, 56124 Pisa, Italy; 3 Multidisciplinary Center of Robotic Surgery, University Hospital of Pisa, 56124 Pisa, Italy; 4 Vascular Surgery Unit, Cisanello Teaching Hospital of Pisa, 56124 Pisa, Italy; 5 Scuola Superiore Sant’Anna of Pisa, 56214 Pisa, Italy; 6 Institute for Medical Science and Technology, University of Dundee, Dundee, DD2 1FD UK

**Keywords:** Artificial intelligence robotic surgery, Machine learning robotic surgery, Deep-learning robotic surgery, Artificial intelligence surgical simulation, Machine learning surgical simulation, Deep-learning surgical simulation

## Abstract

**Background:**

Artificial intelligence (AI) has the potential to enhance patient safety in surgery, and all its aspects, including education and training, will derive considerable benefit from AI. In the present study, deep-learning models were used to predict the rates of proficiency acquisition in robot-assisted surgery (RAS), thereby providing surgical programs directors information on the levels of the innate ability of trainees to facilitate the implementation of flexible personalized training.

**Methods:**

176 medical students, without prior experience with surgical simulators, were trained to reach proficiency in five tasks on a virtual simulator for RAS. Ensemble deep neural networks (DNN) models were developed and compared with other ensemble AI algorithms, i.e., random forests and gradient boosted regression trees (GBRT).

**Results:**

DNN models achieved a higher accuracy than random forests and GBRT in predicting time to proficiency, 0.84 vs. 0.70 and 0.77, respectively (Peg board 2), 0.83 vs. 0.79 and 0.78 (Ring walk 2), 0.81 vs 0.81 and 0.80 (Match board 1), 0.79 vs. 0.75 and 0.71 (Ring and rail 2), and 0.87 vs. 0.86 and 0.84 (Thread the rings 2). Ensemble DNN models outperformed random forests and GBRT in predicting number of attempts to proficiency, with an accuracy of 0.87 vs. 0.86 and 0.83, respectively (Peg board 2), 0.89 vs. 0.88 and 0.89 (Ring walk 2), 0.91 vs. 0.89 and 0.89 (Match board 1), 0.89 vs. 0.87 and 0.83 (Ring and rail 2), and 0.96 vs. 0.94 and 0.94 (Thread the rings 2).

**Conclusions:**

Ensemble DNN models can identify at an early stage the acquisition rates of surgical technical proficiency of trainees and identify those struggling to reach the required expected proficiency level.

**Supplementary Information:**

The online version contains supplementary material available at 10.1007/s00464-021-08999-6.

Surgery is quintessentially a craft medical specialty because the patient outcome depends on the overall quality of the executed operation, and thus, on the surgical skills (cognitive, technical, and non-technical), competence, and perioperative care by the surgeon. Patient outcome ultimately reflects on the quality of surgical training initially proposed by William Halsted [1] and the introduction more than a century later of credentialing and privileges. The Halsted’s training program for surgical residents has been in established use worldwide and remained virtually unchanged for nearly a century until the advent of minimal access surgery (MAS), which posed several challenges on the new skills surgeons have to acquire to perform operations competently and safely by this approach. These skills are perceptual, visuo-spatial, psychomotor, and cognitive [1].

Virtual reality (VR) simulators were developed for the training of basic skills first for laparoscopic surgery and, subsequently, for robot-assisted surgery (RAS) [1]. They enable tracking of trainees’ performances by using built-in algorithms. A seminal randomized control trial (RCT) conducted at Yale University demonstrated that surgical residents trained until proficiency on a VR for laparoscopic surgery completed faster and made five times fewer errors during laparoscopic cholecystectomy on real patients than a control group following traditional apprenticeship training [2].

The level of technical skills for MAS varies greatly among surgeons as shown in a study involving all Michigan hospitals demonstrating that operations performed by highly skilled surgeons had fewer postoperative complications and lower reoperation rates [3].

During the last decade, artificial intelligence (AI) has been applied to several medical specialties, e.g., radiology and dermatology for image analysis. Some studies have indicated that surgical clinical practice stands to gain from the current explosion of AI [4, 5]. In this respect, surgical science has recently adopted a data-driven approach to improve the quality and efficiency of operations through acquisition, analysis, and modeling of data [6]. This approach represents a paradigm shift in surgical clinical practice, including surgical education and training.

Non-linear machine learning (ML) algorithms, especially deep learning (a subclass of ML based on neural networks), have the potential to predict the proficiency-gain curves for operative surgery for two reasons. First, there is no linear relationship between number of attempts and proficiency gain for most surgical trainees. An established method to monitor proficiency-gain curve in surgery is cumulative summation (CUSUM). This describes performance of trainees as a series of incremental or decremental scores, depending on failure or success [7]. However, CUSUM cannot predict important data on trainees’ progress toward efficient and safe execution, e.g., training time and the number of attempts needed to reach the required competence level. Second, proficiency-gain curves vary substantially among individuals as they reflect individual levels of innate technical aptitude [8]. According to the results of a Delphi survey involving surgical program directors in Canada, there is a group of residents, ranging from 5 to 15%, who have trouble in reaching technical competence [9]. In this regard, the demonstration of the robustness of ML models to predict the different rates of proficiency acquisition of residents would be beneficial for the early detection of trainees struggling to achieve the required level of technical skills. Overall, the early identification of proficiency acquisition patterns could enable surgical program directors to customize the teaching of technical skills on individual basis. This would have implications at the level of curriculum planning by estimating in advance the length of training, the time assigned to trainers, and costs in competence-based surgical training curricula.

Despite the potential of ML models for these purposes, the supporting published evidence is limited to a retrospective study on VR for laparoscopic surgery [10]. In this study on 15 medicals students, ML models reached an accuracy of 72.0% and 89.0% to predict, respectively, the number of attempts to reach proficiency and the final performance at the VR simulator [10].

In the present study, we have applied deep neural networks (DNN) for the assessment of progress in surgical skills acquisition. More specifically, we designed ensemble DNN models to identify a threshold, corresponding to the number of attempts needed to predict the training time, and the number of attempts needed to reach proficiency in a group of medical students without prior experience in surgical training. The medical undergraduate students performed exercises on a VR for RAS. The working hypothesis underpinning the study was that DNN models would provide more robust and accurate prediction than other ML algorithms.

## Methods

### Tests procedure

Undergraduate medical students of the University of Pisa (Italy) were recruited by an open non-remunerated call. They had no prior experience with surgical simulation. All recruited students were trained on the at dV-Trainer VR simulator (Mimic, Seattle, WA, United States), which replicates the master console of da Vinci robotic surgical system.

**Fig. 1:**
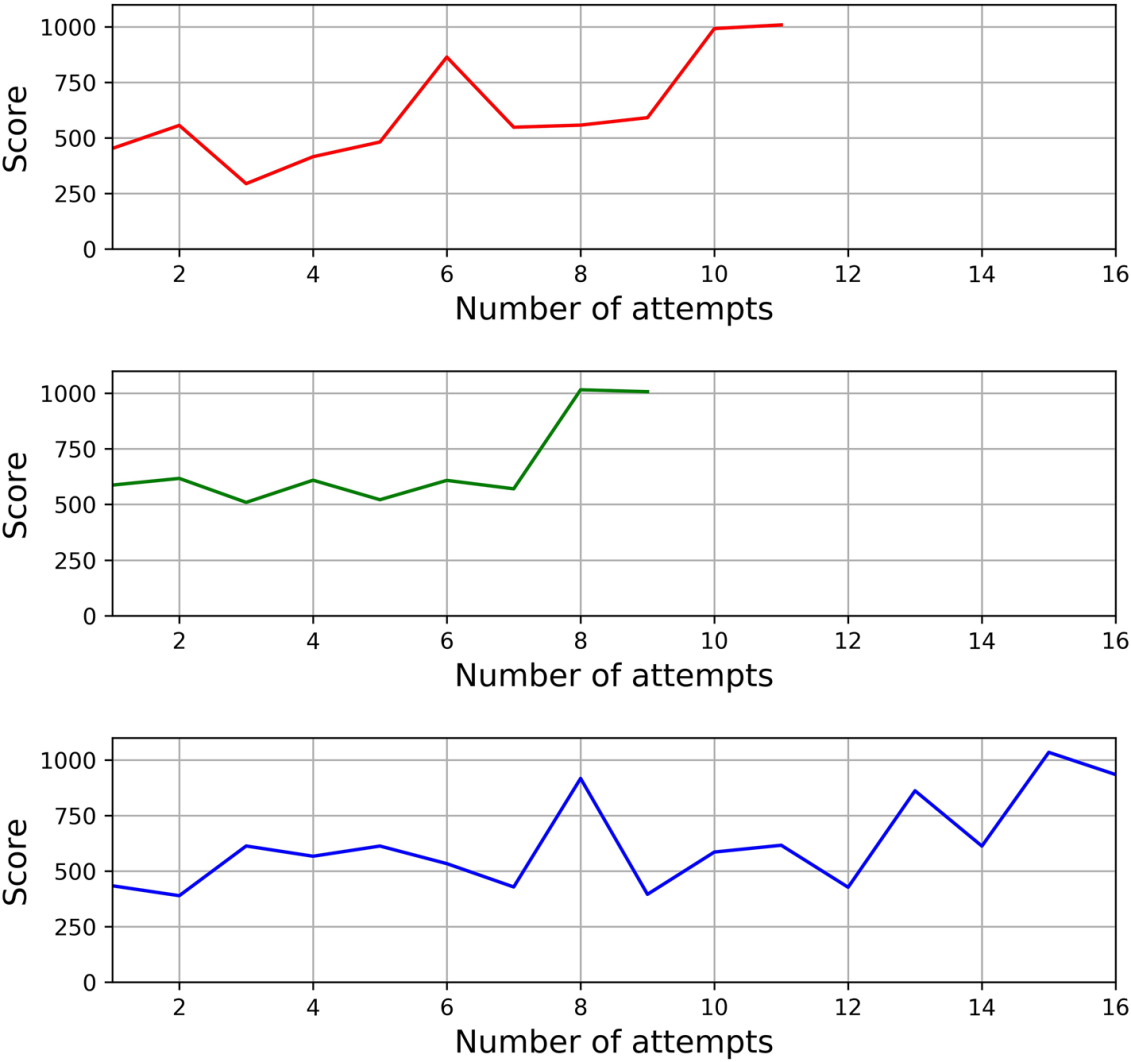
Examples of proficiency-gain curves for RR2 tasks

Participants were allowed ten days to complete successfully the following five exercises on the dV-Trainer: (i) Peg board 2 (PB2), (ii) Ring walk 2 (RW2), (iii) Match board 1 (MB1), (iv) Ring and rail 2 (RR2), and (v) Thread the rings 2 (TR2). The participants had to reach proficiency, defined as ‘performing correctly the exercise with green levels in all metrics twice consecutively,’ before moving to the next exercise. The second execution was required to exclude chance. Examples of proficiency-gain curves for RR2 task are shown in Fig. 1. A tutor was present to provide all participants with instructions on the set up, ergonomics of simulator console, navigation through software menus, effective use of camera, and clutch pedals. Institutional Review Board was not required for this study.

### Data sources

The development of DNN and other ML models was based on data collected at EndoCAS surgical training center of the University of Pisa. These data include medical undergraduates who had been trained to proficiency on a VR simulator for RAS from June 2016 to March 2017. This dataset was enlarged with data from new cases from January to February 2020. Part of the dataset had been published in a previous study from our group, specifically designed to determine the capability of the VR simulator to identify and quantify the size of three medical undergraduate groups with average, high and low innate aptitudes for manipulative skills [8].

### Development of the models

**Fig. 2:**
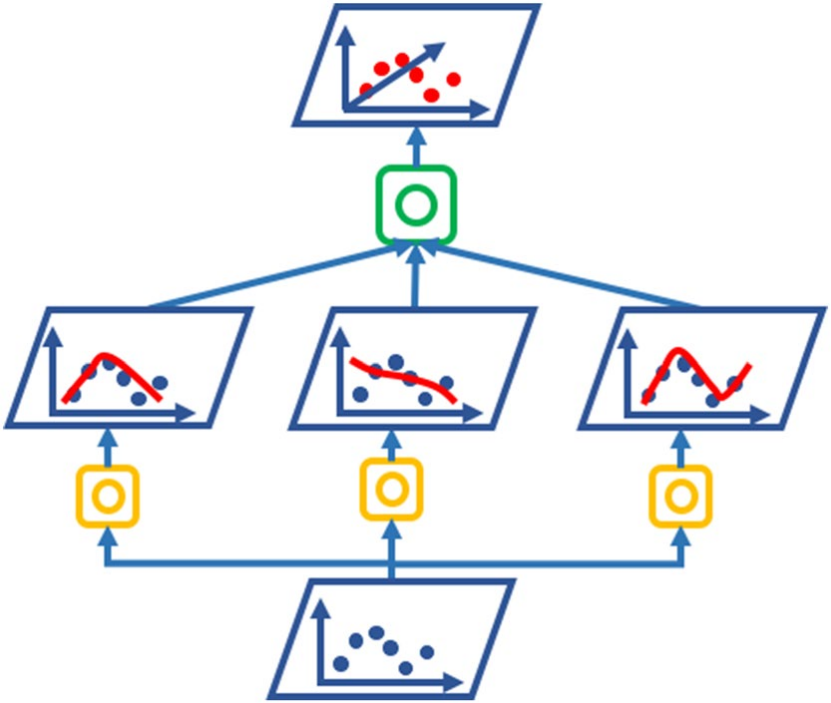
Stacking DNN: data are used to train initial models (orange boxes). Their predictions are used to train the meta-learner (green box). Adapted from [11]

The research was framed as a regression task for supervised learning with training time and number of attempts labeled as target variables. The scores of each attempt on the VR simulator represented the features. Scatter plots confirmed non-linearity between features and target variables. Boxplots revealed the presence of outliers which were included to maintain a large range of proficiency-gain curves. Ensemble AI models were then applied to the dataset. These represent a family of aggregated predictors capable of providing higher accuracy than the individual predictors [11]. Examples of ensemble AI models are random forests, and gradient boosted regression trees (GBRT). In this study, DNN ensemble models were designed. In the stacking configuration of ensemble models, individual models are trained on a training set and their predictions used as input to train a meta-learner (blender), which provides the final prediction on the test data set. The process is depicted in in Fig. 2. More details on the description and implementation of the DNN ensemble models are reported in the Appendix.

The dataset was split randomly into training and test sets in a ratio of 80:20. A hold-out set was used as validation set from the training set in a ratio of 80:20. Grid search cross validation was performed on random forests and GBRT to select the best hyperparameters on the training dataset. Ensemble learning models were then compared with a conventional ML model as reference, i.e., Kernel support vector machine (SVM). Keras with Tensor Flow (version 2.0) as backend and scikit-learn (version 0.22) were used for data analysis.

## Results

A total of 176 medical students completed their training until they reached proficiency. The students comprised 81 (46.0%) males, and 95 females (54.0%), with a mean age of 23.5 (± 3.0) years. They were in the following undergraduate years: UGY1 = 22, UGY2 = 38, UGY3 = 49, UGY4 = 27, UGY5, and 39 UGY6 = 39. The number of participants assessed over the attempts in each task is shown in Table 1. The training time and number of attempts to reach proficiency are reported in Table 2. Training time includes the net execution time of each attempt but excludes the loading time of the exercises and time to provide medical students with feedback.

**Table 1:** Number of medical students assessed during attempts in each task

	Attempt 1	Attempt 2	Attempt 3	Attempt 4	Attempt 5	Attempt 6	Attempt 7	Attempt 8	Attempt 9	Attempt 10	More than 10 attempts
PB2	161	161	157	128	95	65	45	33	20	15	12
RW2	170	170	140	100	74	58	46	27	14	9	6
MB1	172	172	103	67	40	27	18	10	9	7	4
RR2	157	157	145	119	88	73	64	56	40	32	22
TR2	165	165	81	58	40	25	16	13	9	8	6

**Table 2:** Training time and number of attempts expressed as median (range) for all medical students

	Training time (s)	Number of attempts
PB2	883.0 (229.1–3438.1)	5 (2–16)
RW2	747.8 (229.5–2922.2)	4 (2–14)
MB1	794.2 (322.4–4535.9)	3 (2–14)
RR2	1518.8 (418.9–5999.9)	3 (2–22)
TR2	439.6 (217.3–2996.1)	2 (2–14)

### Performances of the DNN ensemble models

The ensemble DNN models obtained the highest accuracy on the same attempt for both target variables: five for PB2, three for RW2 and MB1, four for RR2, and two for TR2, as reported in Tables 3 and 4. The dataset size for each task was 95 for PB2, 140 for RW2, 103 for MB2, 119 for RR2, and 165 for TR2. The ensemble DNN models achieved the following accuracy values on training time: 0.84 for PB2, 0.83 for RW2, 0.81 for MB1, 0.79 for RR2, and 0.87 for TR2. Regarding the accuracy for number of attempts, DNN ensemble achieved: 0.87 for PB2, 0.89 for RW2, 0.91 for MB1, 0.89 for RR2, and 0.96 for TR2. Analysis of features selection for both target variables revealed that ensemble DNN models achieved the highest accuracy by reducing the number of features in RW2 and MB1 task (two out of three features), and RR2 (three out of four). By contrast, ensemble DNN needed all features in the other tasks, respectively, five for PB2 and two for TR2.

**Table 3:** Accuracy values (r2) of ensemble DNN compared with other ML models to predict training time

	Best attempt	Dataset size	Meta-leaner (ensemble DNN)	Random forests	Gradient boosted regression trees	Kernel SVM
PB2	5	95	0.84	0.70	0.77	0.70
RW2	3	140	0.83	0.79	0.78	0.79
MB1	3	103	0.81	0.81	0.80	0.70
RR2	4	119	0.79	0.75	0.71	0.69
TR2	2	165	0.87	0.86	0.84	0.83

**Table 4:** Accuracy values (r2) of ensemble DNN compared with other ML models to predict number of attempts

	Best attempt	Dataset size	Meta-leaner (ensemble DNN)	Random forests	Gradient boosted regression trees	Kernel SVM
PB2	5	95	0.87	0.86	0.83	0.69
RW2	3	140	0.89	0.88	0.89	0.83
MB1	3	103	0.91	0.89	0.89	0.87
RR2	4	119	0.89	0.87	0.83	0.72
TR2	2	165	0.96	0.94	0.94	0.88

### Comparison with other ML models

Data of kernel SVM, a non-ensemble algorithm used as reference of conventional ML algorithms, are also reported. A comparison on accuracy scores of ensemble learning (DNN vs random forests and GBRT) and kernel SVM is outlined in Tables 3 and 4 for training time and number of attempts. All these algorithms reached their highest score in the same attempt as ensemble DNN models for both the target variables. Ensemble DNN outperformed the other algorithms in all instances, with the exception of prediction of training time for MB1 where ensemble DNN and random forests achieved the same accuracy (0.81), and prediction of number of attempts for RW2 where ensemble DNN and GBRT reported the same accuracy (0.89). For feature selection, the same number of features as ensemble DNN was achieved, with the exception of RR2 where the DNN model needed two features for training time compared to three by the other algorithms.

## Discussion

Uptake of AI has spread rapidly to many fields, including medicine. Deep-learning models have been shown to outperform humans in disease detection, e.g., pneumonia from chest X-rays [4]. The current study used non-linear algorithms, i.e., DNN, to demonstrate how these models can detect the proficiency-gain curves of trainees early and correctly in a large group of medical students trained and assessed by a VR simulator for RAS. The results confirmed that DNN models reached a higher accuracy than other ensemble ML algorithms (random forests and GBRT).

The only previously published report on ML for prediction of proficiency-gain in surgery reported an accuracy of 0.72 on the number of attempts to reach proficiency during simulation-based training for laparoscopy [10]. However, this study was limited to 15 medical students, some of whom were assessed by a VR simulator while the others on a physical simulator for laparoscopy. Additionally, each participant was evaluated only on one task [10]. In sharp contrast, the present study assessed a larger number of medical students (ranging from 95 to 165 depending on the exercise) trained on five component surgical tasks. The study has demonstrated that DNN predicted the number of attempts to reach proficiency with higher accuracy than the previously reported study. The accuracy of DNN ranged from 0.87 for PB2 (the task with the highest threshold equivalent to five attempts) to 0.96 for TR2, the task with the lowest threshold (equivalent to two attempts). The developed DNN models also predicted the training time to proficiency with an accuracy ranging from 0.79 for RR2 (the exercise taking the longest time to complete training) to 0.87 for TR2. Additionally, the study confirmed the high capability of DNN to learn complicated training patterns with much higher accuracy than ensemble tree-based models (random forest and GBRT), and conventional non-linear ML algorithms such as kernel SVM.

To date, there have been few published studies on AI applied to training in RAS. Moreover, they did not assess the proficiency-gain curves. One study applied support vector machine to define the stylistic behavior of 14 participants with different level of experience during the execution of two tasks for three times on a VR simulator for RAS [12]. Other studies applied complex deep-learning models, e.g., convolutional neural networks and/or recurrent neural networks, for motion analysis of the John Hopkins University Intuitive Surgical Gesture and Skill Assessment Working Set (JIGSAWS), a public dataset with video and kinematic data of eight surgeons performing three RAS tasks (suturing, needle passing, and knot tying) on inanimate models [13]. Another study applied support vector machines to JIGSAWS dataset to predict skills classifications and scores assessed by Objective Structured Assessment of Technical Skill (OSATS) and Global Evaluative Assessment of Robotic Surgery (GEARS) [14].

The present study has two implications for surgical education and training. First, in view of the growing published evidence that there is group of surgical trainees who struggle to reach the required competence level. The first report in this field involved 37 residents trained by a VR for laparoscopy confirming an incidence of 8.1% of subjects who were unable to be trained in the laparoscopic surgical approach [15]. Another study reported that 15.0% of subjects with a low ability to reach proficiency for laparoscopic appendectomy on a simulator [16]. More recently, two studies by our group on 121 and 155 medical students found, respectively, an incidence 11.6% and 11.0% exhibiting a low level of innate ability for RAS [8, 17]. Based on these reports, some surgical training program directors have explored the use of VR simulators as an objective assessment tool of surgical innate aptitude for surgical technical skills to complement the current selection process of candidates applying to recognized surgical training programs [8, 17]. The bottleneck of such a test lies in the extra time and costs required to complete the selection process. In the present study, we report that after few attempts (varying from two for TR2 to five for PB2), DNN models can predict the individual proficiency-gain curves of applicants, thereby significantly reducing the overall selection process.

The methods of implementing AI models reported by this study can be extended to any kind of VR surgical simulation. The availability of the counterpart of our DNN models for laparoscopy would enable a thorough evaluation of innate technical skills for MAS. This test may serve as a robust objective assessment of technical skills to complement the selection of surgical residents. However, surgical residents must acquire competence during training also in other crucial domains for safe execution of surgery such as cognition, decision making, and non-technical skills, e.g., human factors.

Second, the early detection of the skills deficits among low performing trainees would enable provision of feedback to them with consideration of extending their training period to reach surgical competence, i.e., flexible training period would become an integral component of a competence-based surgical curriculum.

This study has some limitations, the most important being the small sample size of training and test sets. The reason for this stems from the long time required in studies acquiring data on surgical proficiency-gain curves. Second, data of our study come from a single center. Since the VR simulator for RAS used in this study is available in many surgical training centers, a larger multi-institutional study would allow to capture a significantly larger range of trainees. A multicenter study would, thus, enable a more robust assessment validation of the predictive accuracy of the developed AI models when applied to external centers. Third, the AI models we have developed are limited to prediction of proficiency during surgical training using a simulator. They cannot be used to predict clinical performance during execution of surgery on real patients.

## Conclusions

The present study is the first to apply DNN models for the early prediction of proficient execution of surgical component tasks during the acquisition of basic skills of RAS. In this respect, it has the potential to usher a new era of surgical training characterized and underpinned by flexible surgical training, based on innate aptitude for manipulative psychomotor skills and tailored to the needs of individual surgical residents. Consequently, the gifted trainees, who will require significantly less time to reach proficiency, should complete this stage of their training sooner than the average ones and qualify for the next phase of training. It will add meaning to the modern emphasis on competence-based surgical curricula.

## Supplementary Information

Below is the link to the electronic supplementary material.

Supplementary file1 (DOCX 17 kb)
