# Supplementary material for: Ensemble deep learning for the prediction of proficiency at a virtual simulator for robot-assisted surgery
Source: Surg Endosc. 2022 Jan 12;36(9):6473–9. doi: 10.1007/s00464-021-08999-6 (PMC9402513; doi:10.1007/s00464-021-08999-6)
Supplement: Supplementary file 1 — Supplementary file1 (DOCX 17 kb) [file 464_2021_8999_MOESM1_ESM.docx]

**Appendix**

**Development of the models**

The research was framed as supervised learning since the training set includes the desired solutions, namely training time and number of attempts to reach proficiency. The scores at each attempt represent the features, while training time and the number of attempts to reach proficiency are the target variables. We used regression to predict these two target variables using the features. Scatter plots showed non-linearity between features and target variables. For this reason, correlation was investigated by means of distance correlation. Boxplots revealed the presence of outliers among features with respect to the targets. Outliers can be removed to prevent overfitting, i.e. when a model performs well in the training set but not as good in the test set. Since the purpose of the study is to predict proficiency-gain curves of surgical trainees, a large range of proficiency gain-curves is essential. For this reason, the outliers were included. We achieved this by transforming data through quantile transformation as this reduces the impact of outliers [1]. Ensemble DNN models were specifically developed for this study.

***Ensemble models of machine learning***

Ensemble models were then applied to the dataset. These represent a family of aggregated predictors capable of providing higher accuracy than the individual predictors, obtaining the so-called wisdom of the crowd [2]. It is assumed that, while the individual models make different errors, ensemble models reduce the overall error rate, thereby achieving higher accuracy [3]. Example of ML ensemble algorithms are random forests combining decision trees to reduce overfitting of training data, and gradient boosted regression trees (GBRT) merging several weak learners into a stronger one [2]. Ensemble learning can be particularly useful to counter a drawback of neural networks, namely the high variance.

In the stacking configuration, individual models are trained on a training set and their predictions on a validation set are used as input to train a meta-learner (blender), which makes the final prediction on the test set. The current study employed DNN dense layers, where each neuron in a hidden layer is connected to all neurons of the next hidden layer [2].

The dataset was split randomly into training and test set in a ratio of 80:20. A hold-out set was used as validation set from the training set in a ratio of 80:20. Grid search cross validation was performed on random forests and GBRT to select the best hyperparameters on the training dataset. The training set and test set for each task were the same for DNN and ML models. Ensemble learning models were then compared with a conventional ML model as reference, using. Kernel SVM.

***Implementation of DNN models***

The individual models forming the ensemble DNN model were optimized using the following strategy. Firstly, He initialization was used during initialization of weights coefficients to avoid the issue of vanishing/exploding gradients leading the backpropagation algorithm of a non-convergent/divergent solution during training DNN models [4]. Rectified Linear Unit (ReLU) was used as activation function. Secondly, dropout was used to avoid overfitting, and Gaussian Noise for data augmentation to improve generalization. Thirdly, to speed up the gradient descent optimization, different algorithms were tested: momentum, Nesterov, and Adaptive moment estimation (Adam). Although for regression purposes, the loss function to estimate the error between prediction and actual value is generally either mean squared error or mean absolute error, we used Huber loss since it is less sensitive to outliers than the mean squared error. Additionally, it converges faster and is more precise than the mean absolute error [2].

***Evaluation of models***

Accuracy was assessed through coefficient of determination r^2^, defined as:

| $r^{2}=1- \frac{SSE}{SST}$ | (1) |
| --- | --- |
| $SSE= \frac{\sum_{i=1}^{n} {(y_{i}-\hat{y_{i}})}^{2}}{n-1}$ | (2) |
| $SST= \frac{\sum_{i=1}^{n} {(y_{i}-\bar{y})}^{2}}{n-1}$ | (3) |

Where y_i_ is the true value, $\hat{y_{i}}$ the predicted value of the target variable, $\bar{y}$ the mean value of the true target variable, and *n* the number of samples. The following procedure was iterated for all tasks:

1. Accuracy was assessed from the second attempt onwards to identify obtain the highest accuracy possible.
2. Features selection, based on univariate statistics, was applied to consider only the most significant features. Keras with Tensor Flow (version 2.0) as backend and scikit-learn (version 0.22) were used for data analysis.

**References**

1. [online]: https://scikit-learn.org/stable/modules/generated/sklearn.preprocessing.QuantileTransformer.html (last access on November 22, 2021.
2. Géron A. Hands-on Machine Learning with Scikit-Learn, Keras & TensorFlow, 2nd Edition. O’Reilly Media, 2019.
3. Wang M, Cang Z, Wei GW. A topology-based network tree for the prediction of protein–protein binding affinity changes following mutation. Nat Mach Intell 2020; 2: 116–123.
4. He K, Zhang X, Ren S, Sun J. Delving Deep into Rectifiers: Surpassing Human-Level Performance on ImageNet Classification. Proceedings of the 2015 IEEE International Conference on Computer Vision 2015: 1026–34.
